# Supplementary material for: Community-level impacts of spatial repellents for control of diseases vectored by Aedes aegypti mosquitoes
Source: PLoS Comput Biol. 2020 Sep 25;16(9):e1008190. doi: 10.1371/journal.pcbi.1008190 (PMC7541056; doi:10.1371/journal.pcbi.1008190)
Supplement: S1 Text — (DOCX) [file pcbi.1008190.s001.docx]

S1 Text

**Materials and Methods**

***Analysis of blood feeding experiments***

| $a_{p}(dose)=\frac{1}{e^{\beta_{p,control}+\beta_{p,dose}}}$ | (S1) |
| --- | --- |
| and |  |

Mosquitoes in the blood-feeding experiment could have experienced three different outcomes: partially blood-fed, fully blood-fed, or not blood-fed. We consider the biting rate *a* referred to previously as the sum of the rates of partial and full blood feeding, *a_p_* and *a_f_*, respectively. We modeled these rates as functions of the dose of transfluthrin, *x_dose_*, as

| $a_{f}(dose)=\frac{1}{e^{\beta_{f,control}+\beta_{f,dose}}}$ | (S2) |
| --- | --- |

We assumed that a mosquito can potentially take multiple partial blood meals, with a single partial blood meal having no impact on the probability of taking another partial blood meal or a full blood meal. However, we assume that full blood meals are absorbing states, in the sense that a full blood meal prevents any further blood feeding thereafter within the timeframe of the experiment.

Under these assumptions, the expected number of partial blood meals after time *t* given *a_p_*(*dose*) is *a_p_*(*dose*)*t*, provided that blood feeding follows a Poisson process. This results in the probability of *k* partial blood meals after time *t* being described by a Poisson distribution with rate parameter *a_p_*(*dose*)*t*. Similarly, the probability of a full blood meal to have occurred by time *t* is the complement of the probability that no full blood meal has occurred by that time; *i.e*., $1-e^{-a_{f}\left( dose \right)t}.$

The number of fully blood-fed mosquitoes observed in the experiment includes both mosquitoes that were fully engorged after one meal and those that became fully engorged after multiple partial blood meals. Therefore, the probability for a mosquito to be partially blood-fed is the probability of the intersection of *k*_p_ > 0 and *k*_f_ = 0, which is

| $Pr(B_{p})=\left( 1-Pr \left( k_{p}=0 \right) \right)Pr\left( k_{f}=0 \right).$ | (S3) |
| --- | --- |

The probability of observing a mosquito that is not blood-fed equals

| $Pr(B_{0})=Pr \left( k_{p}=0 \right)Pr\left( k_{f}=0 \right),$ | (S4) |
| --- | --- |

and the probability of observing a fully blood-fed mosquito is

| $Pr\left( B_{f} \right)=1-\Pr\left( B_{p} \right)-Pr(B_{0}).$ | (S5) |
| --- | --- |

| $L=P_{multinom}(\beta_{p,control}\beta_{p,low}\beta_{p,high}\beta_{f,control}\beta_{f,low}\beta_{f,high}\vert N_{p},N_{f},N_{0}).$ | (S6) |
| --- | --- |

Using the probabilities in eqns. S3-5 and the observed number of mosquitoes (*N*) in each feeding category, we calculated the likelihood of the model parameters given the observed data as

We maximized this likelihood using the *bbmle* package [1] in R to obtain best-fit estimates of the coefficients *β_p_*_,control_, *β_p_*_,dose_, *β*_,control_ , and *β_f_*_,dose_ in eqns. S1 and S2 that describe the dosage effects of the SR on the biting rates *a_p_* and *a_f_*.

We also explored two models that captured more complex biting rates over time. 1: As mosquitoes were starved prior to the experiment, a substantial proportion of mosquitoes fed shortly after the start of the experiment *Pr*(*B*_direct_). A proportion *p_full_* of these direct feeds would result in a fully blood fed mosquito and (1- *p_full_*) in a partially blood fed one. Under this model, *Pr*(*B_f_*) follows from the union of directly taking a full blood meal (*Pr*(*B_direct_*)**p_full_*) or becoming fully blood fed later (as described above). The same logic holds for *Pr*(*B_p_*). 2: For the second model expansion, we additionally assume a non-zero probability for mosquitoes to never feed after exposure to the product *Pr*(*B_never_*). Either biting probabilities are modeled as the intersect of (1- *Pr*(*B_never_*)) and the aforementioned formulations of full and partial biting rates. Model alternatives are compared using Akaike Information Criteria (AIC) [2].

***Analysis of longevity experiments***

Conditional on surviving day one, the data consist of a set of interval- and right-censored time-to-event data [3]. Let Ψ be a random variable representing the time until death, *f*(Ψ=*t*) its probability density function (pdf), and *F*(Ψ=*t*) its cumulative distribution function (cdf). The complement of the cdf is the survival function *S*(*t*), defined mathematically as

| $S\left( t \right)=\Pr\left\{ \Psi\geq t \right\}=1-F\left( t \right)=\int_{t}^{\infty} f\left( x \right)dx ,$ | (S7) |
| --- | --- |

which describes the probability that death has not yet occurred by time *t*. A related characterization of the distribution of Ψ, and fundamental to survival analysis, is the hazard function, *λ*(*t*) [3]. The hazard denotes the instantaneous rate at which death occurs and is described as the probability that death occurs between time *t* and time *t*+*dt*, given that *dt* is small and death has not occurred before t, taking the form

| $\lambda\left( t \right)=\lim_{dt\to\infty} \frac{Pr\{t\leq\Psi<t+dt\vert\Psi\geq t\}}{dt}.$ | (S8) |
| --- | --- |

The hazard can be written as the ratio of the joint probability that Ψ is in the interval [*t*, *t*+*dt*] and that Ψ > *t*

| $\lambda\left( t \right)=\frac{f(t)}{S(t)}.$ | (S9) |
| --- | --- |

To estimate the effect of chemical dosage on the hazard over time, we fitted different survival models to the time-to-event data collected in the laboratory experiments. A specific feature of these data is censoring; for some mosquitoes, death has not yet occurred by the end of the study and thus we only know that Ψ exceeds the observation time. An important assumption here is that this censoring is independent of the probability of an event to occur.

For uncensored mosquito *i*, we can express the likelihood of a given hazard model conditional on an observed event time Ψ = *t*_i_ as the product of *S*(*t*) and *λ*(*t*) as

| $L_{i}=f(t_{i})=S\left( t_{i} \right)\lambda(t_{i}).$ | (S10) |
| --- | --- |

For censored observations, the likelihood function only reflects that Ψ exceeds *t*_i_

| $L_{i}=S\left( t_{i} \right).$ | (S11) |
| --- | --- |

We can write this into a single expression

| $L=\prod_{i=1}^{n} L_{i}=\prod_{i} {\lambda(t_{i})}^{d_{i}}S(t_{i}),$ | (S12) |
| --- | --- |

where *d*_i_ is the event indicator and takes a value of 1 if the event has occurred before the end of the observation time and 0 otherwise.

The specific form of the hazard model that we seek to estimate is a function of the transfluthrin dose *x*_dose_, expressed at time *t* as

| $\lambda_{i}\left( t \vert dose \right)=\lambda_{0}(t)e^{\beta_{dose}},$ | (S13) |
| --- | --- |

where *λ*_0_(*t*) is the baseline hazard for individuals with dose equal to zero (*i.e*., the control group) and $e^{\beta_{dose}}$ is the relative hazard associated with a specific dosage, which is assumed to be stable over time. Such proportional hazard (PH) models allow for a distinct separation of the effect of time from the effect of the covariate. This becomes clearer when we express the model in terms of the log of the hazards, finding a simple additive model $\ln\left( \lambda_{i}\left( t | dose \right) \right)=\alpha_{0}\left( t \right)+\beta_{dose}.$ We will assume that PH holds in this analysis; *i.e*., that the relative effects of the products do not change over time. One can verify this assumption by performing a log transformation on the survival data (S4 Fig) and assessed whether, under this transformation, the curves fitted to observations were parallel across dosages. Waning of the strong treatment effect briefly after exposure resulted in departures from PH at early timepoints. In reality, exposure to treatment will likely be more gradual and long-lasting than in this experimental design, with effects more akin to later time points. Therefore, we feel PH to be a reasonable assumption.

The shape of the baseline hazard *λ*_0_(t) affects the results of the final proportional hazards model. Under the assumption that the distribution of survival times is the result of a continuous-time stochastic process, one can use parametric survival models to describe *λ*_0_(*t*) [3]. We examined the performance of five candidate models with different assumptions about *λ*_0_(*t*) (S4 Fig). The exponential model assumes a stable baseline hazard *λ*(*t*) = *λ*. Alternatively, the Weibull model allows *λ*(*t*) to monotonically increase or decrease over time: $\lambda\left( t \right)=v\lambda\left( \lambda t \right)^{v-1}$. The gamma distribution too allows for *λ*(*t*) to monotonically increase or decrease. There is no closed form expression for the hazard function of the gamma distribution. Whereas the exponential model describes the waiting time until the first event occurs in a Poisson process, the gamma describes the waiting time until *k* events occur. For comparable rate parameters *k* or *v* (S4 Fig), the hazard described by the Weibull distribution decreases more rapidly than in a gamma distribution. The lognormal model allows for an initial increase in *λ*(t) to be followed by a decrease as time progresses. Lastly, the generalized gamma distribution is an extension of the gamma distribution with an added scale parameter that allows for increased flexibility in *λ*(*t*). The gamma (*v* =1), Weibull (*κ*=1), lognormal ($\kappa\to inf$), and exponential (*κ*=1 and *v* =1) models are all special, nested cases of the generalized gamma distribution. We used the Akaike Information Criterion [2] to select the best model, thereby accounting for different numbers of parameters across models. The models were fitted and assessed using the *survival* package 2.27-7 [4] and the *flexsurv* package [5] in R 3.2.3 [6].

References

1.     Bolker B. (2016) Package ‘bbmle’. .

2.     Akaike H. (1973) Information theory and an extension of the maximum likelihood principle. : 267-281.

3.     Woodward M. (2014) Epidemiology: Study design and data analysis. : CRC Press.

4.     Therneau T. (2013) A package for survival analysis in S. R package version 2.37-4. URL Http://CRAN.R-Project.Org/Package= Survival.Box 980032: 23298-20032.

5.     Jackson CH. (2016) Flexsurv: A platform for parametric survival modelling in R. Journal of Statistical Software 70: 1-33.

6.     Team RC. (2014) R: A Language and Environment for Statistical Computing.R Foundation for Statistical Computing, Vienna, Austria.2013 .

7.     ten Bosch QA, Castro-Llanos F, Manda H, Morrison AC, Grieco JP, et al. (2018) Model-based analysis of experimental data from interconnected, row-configured huts elucidates multifaceted effects of a volatile chemical on aedes aegypti mosquitoes. Parasites & Vectors 11: 365.
