# Supplementary material for: Community-level impacts of spatial repellents for control of diseases vectored by Aedes aegypti mosquitoes
Source: PLoS Comput Biol. 2020 Sep 25;16(9):e1008190. doi: 10.1371/journal.pcbi.1008190 (PMC7541056; doi:10.1371/journal.pcbi.1008190)
Supplement: S1 Fig — (A) exponential, (B) Weibull, (C) lognormal, and (D) gamma models. The dashed lines depict the Kaplan-Meier curves at associated dosages. (DOCX) [file pcbi.1008190.s002.docx]

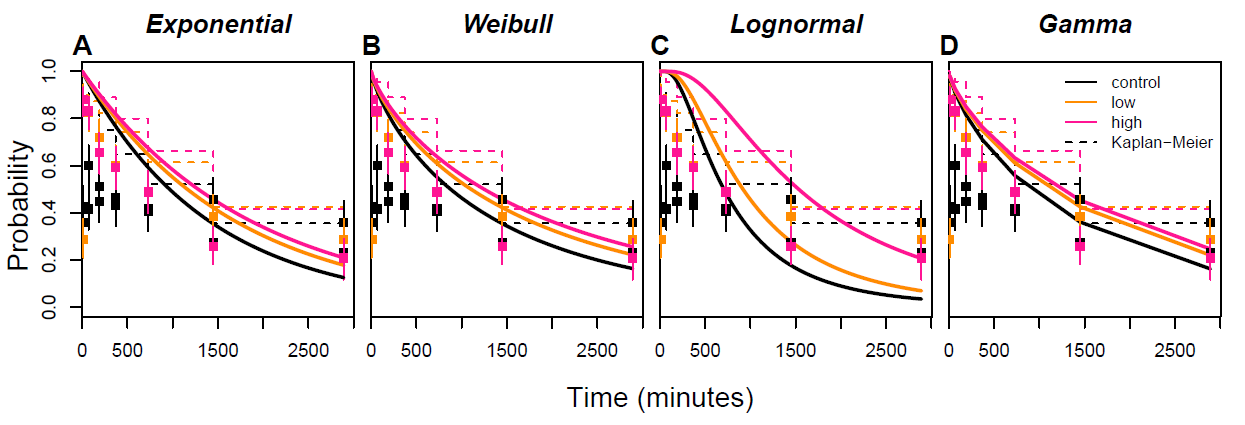


**S1 Fig. Estimated dose effects of an experimental SR product containing transfluthrin on mosquito blood feeding.** (A) exponential, (B) Weibull, (C) lognormal, and (D) gamma models. The dashed lines depict the Kaplan-Meier curves at associated dosages.
