# Supplementary material for: Community-level impacts of spatial repellents for control of diseases vectored by Aedes aegypti mosquitoes
Source: PLoS Comput Biol. 2020 Sep 25;16(9):e1008190. doi: 10.1371/journal.pcbi.1008190 (PMC7541056; doi:10.1371/journal.pcbi.1008190)
Supplement: S2 Fig — (A,D,G) fully blood-fed, (B,E,H) partially blood-fed, and (C,F,I) unfed Aedes aegypti mosquitoes under control (A,B,C), low (D,E,F), and high (G,H,I) dosage regimens. We distinguish a model that i) assumes constant biting rates (solid), ii) as i but preceded by a proportion of mosquitoes feeding directly at the start of the experiment (dashed), and iii) as ii but with a proportion of mosquitoes that will never feed altogether (dotted). Squares denote the observed data for control (black), low (orange), and high dosage regimen (pink). Bars denote the corresponding binomial 95%-confidence intervals. (DOCX) [file pcbi.1008190.s003.docx]

**S2 Fig. Effect estimates of an experimental SR product containing transfluthrin on the probability of blood feeding over time for different model alternatives.** (A,D,G) fully blood-fed, (B,E,H) partially blood-fed, and (C,F,I) unfed *Aedes aegypti* mosquitoes under control (A,B,C), low (D,E,F), and high (G,H,I) dosage regimens. We distinguish a model that i) assumes constant biting rates (solid), ii) as i but preceded by a proportion of mosquitoes feeding directly at the start of the experiment (dashed), and iii) as ii but with a proportion of mosquitoes that will never feed altogether (dotted). Squares denote the observed data for control (black), low (light orange), and high dosage regimen (dark orange). Bars denote the corresponding binomial 95%-confidence intervals.
