# Supplementary material for: Community-level impacts of spatial repellents for control of diseases vectored by Aedes aegypti mosquitoes
Source: PLoS Comput Biol. 2020 Sep 25;16(9):e1008190. doi: 10.1371/journal.pcbi.1008190 (PMC7541056; doi:10.1371/journal.pcbi.1008190)
Supplement: S3 Fig — (A) exponential, (B) Weibull, (C) lognormal, (D) gamma, and (E) generalized gamma models. The dashed lines depict the Kaplan-Meier curves at associated dosages (control, low, high). (DOCX) [file pcbi.1008190.s004.docx]

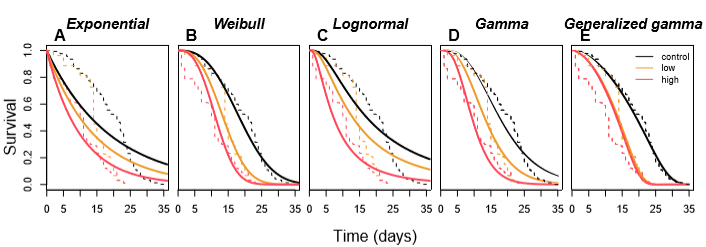


**S3 Fig. Estimated dose effects of an experimental SR product containing transfluthrin on mosquito longevity.** (A) exponential, (B) Weibull, (C) lognormal, (D) gamma, and (E) generalized gamma models. The dashed lines depict the Kaplan-Meier curves at associated dosages (control, low, high).
