# Supplementary material for: Community-level impacts of spatial repellents for control of diseases vectored by Aedes aegypti mosquitoes
Source: PLoS Comput Biol. 2020 Sep 25;16(9):e1008190. doi: 10.1371/journal.pcbi.1008190 (PMC7541056; doi:10.1371/journal.pcbi.1008190)
Supplement: S4 Fig — The proportional hazards assumption holds for dosage regimen that are parallel to each other when plotted with these transformations. (DOCX) [file pcbi.1008190.s005.docx]

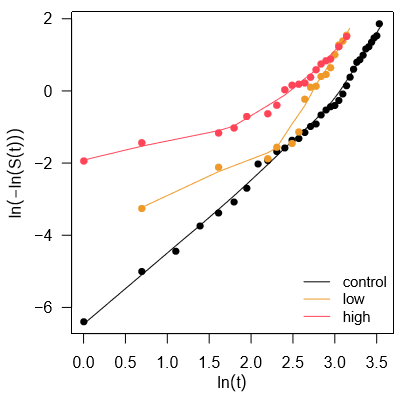


**S4 Fig. Proportional hazard test for longevity data conditioned on first day survival.** The proportional hazards assumption holds for dosage regimen that are parallel to each other when plotted with these transformations.
