# Supplementary material for: Community-level impacts of spatial repellents for control of diseases vectored by Aedes aegypti mosquitoes
Source: PLoS Comput Biol. 2020 Sep 25;16(9):e1008190. doi: 10.1371/journal.pcbi.1008190 (PMC7541056; doi:10.1371/journal.pcbi.1008190)
Supplement: S5 Fig — (A) repellency (decreased entry) and (B) expellency (increased exit) during exposure to control, low (0.0025 g/m2), and high (0.005g/m2) dose regimen. (DOCX) [file pcbi.1008190.s006.docx]

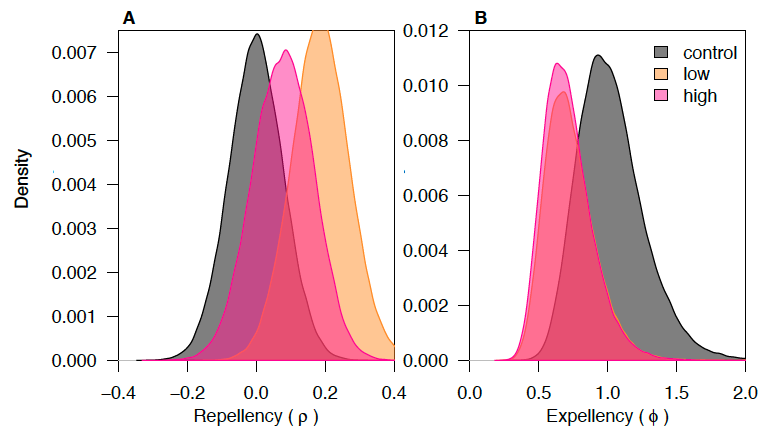


**S5 Fig. Posterior estimates of effects of an experimental SR product containing transfluthrin.** (A) repellency (decreased entry) and (B) expellency (increased exit) during exposure to control, low (0.0025 g/m2), and high (0.005g/m2) dose regimen.
