# Supplementary material for: Community-level impacts of spatial repellents for control of diseases vectored by Aedes aegypti mosquitoes
Source: PLoS Comput Biol. 2020 Sep 25;16(9):e1008190. doi: 10.1371/journal.pcbi.1008190 (PMC7541056; doi:10.1371/journal.pcbi.1008190)
Supplement: S6 Fig — (A) extrinsic incubation period from 3 to 33 days, (B) transmission probability from mosquito to human from 0.01 to 1, (C) as B but from human to mosquito, (D) human infection prevalence from 1 to 99%, (E) the duration of the gonotrophic cycle from 1 to 14 days, (F) the baseline biting rate from 0.2 to 10, (G) the baseline mosquito mortality rate from 0.025 to 2.5, (H) mortality rate during transit relative to indoor mortality rate from 0.1 to 10, (I) average time spent in an untreated house from 0.05 to 5 days, and (J) the proportion of time a transit event takes relative to the baseline residence time, from 0.1 to 10. Across all panels, yellow depicts low values and dark red signifies high values. (DOCX) [file pcbi.1008190.s007.docx]

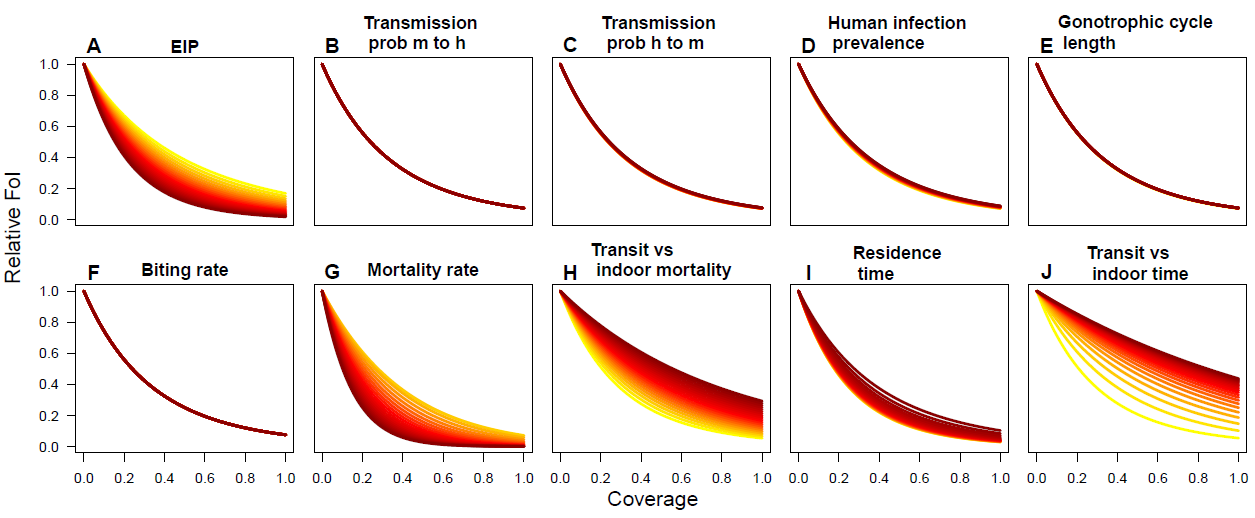


**S6 Fig. Sensitivity of relative force of infection (FoI) estimates to the baseline parameters as a function of population coverage.** (A) extrinsic incubation period from 3 to 33 days, (B) transmission probability from mosquito to human from 0.01 to 1, (C) as B but from human to mosquito, (D) human infection prevalence from 1 to 99%, (E) the duration of the gonotrophic cycle from 1 to 14 days, (F) the baseline biting rate from 0.2 to 10, (G) the baseline mosquito mortality rate from 0.025 to 2.5, (H) mortality rate during transit relative to indoor mortality rate from 0.1 to 10, (I) average time spent in an untreated house from 0.05 to 5 days, and (J) the proportion of time a transit event takes relative to the baseline residence time, from 0.1 to 10. Across all panels, yellow depicts low values and dark red signifies high values.
