# Supplementary material for: Community-level impacts of spatial repellents for control of diseases vectored by Aedes aegypti mosquitoes
Source: PLoS Comput Biol. 2020 Sep 25;16(9):e1008190. doi: 10.1371/journal.pcbi.1008190 (PMC7541056; doi:10.1371/journal.pcbi.1008190)
Supplement: S7 Fig — Posterior distributions of model parameters fitted to experimental data for the baseline (gray), low (orange) and high (pink) transfluthrin dosage for the treated hut (subscript T) and huts one or two removed from the treated hut (subscript 1 and 2, respectively). (reproduction from Figure 6 in [22]). Posterior distributions of model parameters fitted to experimental data for the baseline (gray), low (orange) and high (pink) transfluthrin dosage for the treated hut (subscript T) and huts one or two removed from the treated hut (subscript 1 and 2, respectively). (a-c) Rates at which mosquitoes exit the huts. (d) Proportion of movement from H1 (hut directly adjacent to the treatment hut) away from the SR, where the dashed line indicates p1 = 0.5, i.e. no repellency effect. (e-g) Knockdown rates. (h) Loss to follow-up rates. Under this parameterization, the movement rate q i is exactly equal to the product x i / r i. The algorithm was run for 90,000 iterations inclusive of a ‘burn-in’ period of 10,000. (DOCX) [file pcbi.1008190.s008.docx]

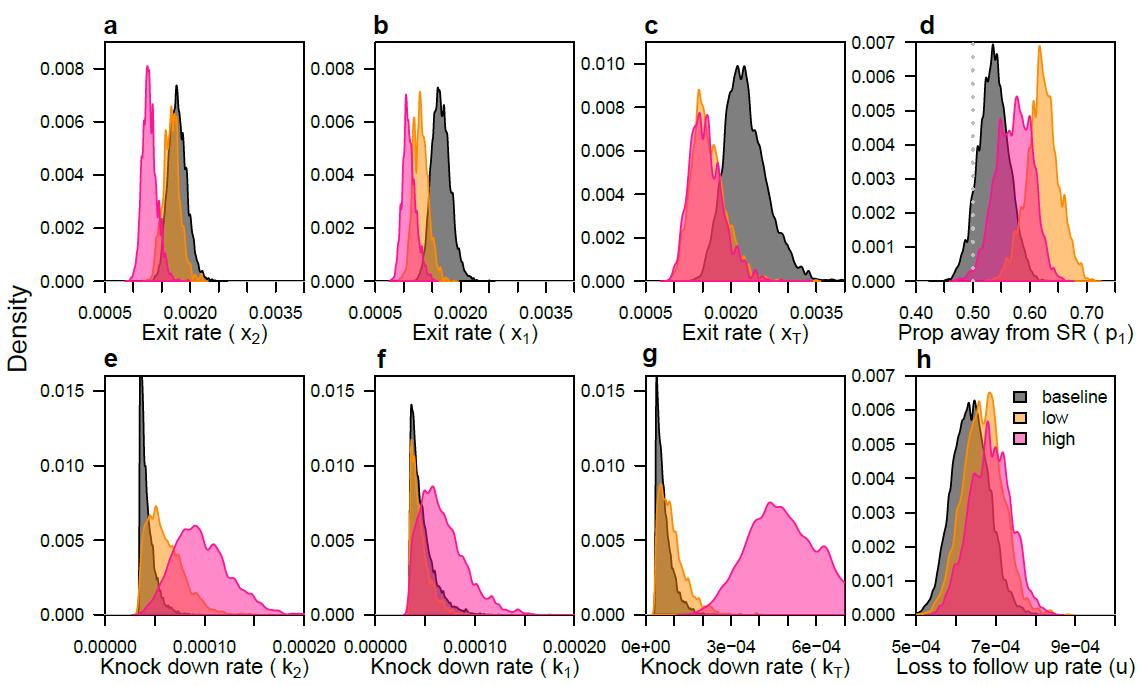


**S7 Fig. Posterior distributions of model parameters fitted to experimental data for the baseline (gray), low (orange) and high (pink) transfluthrin dosage for the treated hut (subscript T) and huts one or two removed from the treated hut (subscript 1 and 2, respectively).**  **(reproduction from Figure 6 in [7]).** Posterior distributions of model parameters fitted to experimental data for the baseline (gray), low (orange) and high (pink) transfluthrin dosage for the treated hut (subscript T) and huts one or two removed from the treated hut (subscript 1 and 2, respectively). (a-c) Rates at which mosquitoes exit the huts. (d) Proportion of movement from H1 (hut directly adjacent to the treatment hut) away from the SR, where the dashed line indicates p1 = 0.5, i.e. no repellency effect. (e-g) Knockdown rates. (h) Loss to follow-up rates. Under this parameterization, the movement rate q i is exactly equal to the product x_i_ / r_i_ . The algorithm was run for 90,000 iterations inclusive of a ‘burn-in’ period of 10,000.
