# Supplementary material for: Community-level impacts of spatial repellents for control of diseases vectored by Aedes aegypti mosquitoes
Source: PLoS Comput Biol. 2020 Sep 25;16(9):e1008190. doi: 10.1371/journal.pcbi.1008190 (PMC7541056; doi:10.1371/journal.pcbi.1008190)
Supplement: S1 Table — (DOCX) [file pcbi.1008190.s009.docx]

**S1 Table. Comparing functional forms of transfluthrin effects on blood feeding (low dosage, 8.4x10^-7^ g/L)**

| Model | Increase in time until bloodfed (%) | 95% confidence interval |
| --- | --- | --- |
| Multinomial model estimate (see methods) | 28 | (13, 43) |
| exponential | 20 | (7, 35) |
| Weibull | 26 | (9, 45) |
| log-normal | 30 | (8, 56) |
| gamma | 24 | (8, 42) |
