# Supplementary material for: Community-level impacts of spatial repellents for control of diseases vectored by Aedes aegypti mosquitoes
Source: PLoS Comput Biol. 2020 Sep 25;16(9):e1008190. doi: 10.1371/journal.pcbi.1008190 (PMC7541056; doi:10.1371/journal.pcbi.1008190)
Supplement: S2 Table — The AIC (Akaike Information Criterium) denotes the model fit, with a lower fit presenting a better fit [46]. (DOCX) [file pcbi.1008190.s010.docx]

**S2 Table. Comparing models on Transfluthrin effects on time until 50% of mosquitoes blood fed (low dosage, 8.4x10^-7^ g/L). The AIC (Akaike Information Criterium) denotes the model fit, with a lower fit presenting a better fit [46].**

| Model | Time until 50% fully blood fed (partial) | |  | | AIC |
| --- | --- | --- | --- | --- | --- |
|  | **control** | **low** | **high** | |  |
| 1:Multinomial model estimate (see methods) | 12 (35) | 18 (34) | | 22 (27) | 3195 |
| 2:1 + proportion direct feeding | 10 (48) | 25 (48) | | 23 (30) | 633 |
| 3:2 + proportion never feeding | 10 (48) | 25 (48) | | 18 (27) | 625 |
