# Supplementary material for: Community-level impacts of spatial repellents for control of diseases vectored by Aedes aegypti mosquitoes
Source: PLoS Comput Biol. 2020 Sep 25;16(9):e1008190. doi: 10.1371/journal.pcbi.1008190 (PMC7541056; doi:10.1371/journal.pcbi.1008190)
Supplement: S3 Table — (DOCX) [file pcbi.1008190.s011.docx]

**S3 Table. Transfluthrin effects on mortality by survival model (low dosage, 8.4x10^-7^ g/L)**

| Model | ϕ | 95% confidence interval |
| --- | --- | --- |
| exponential | 0.75 | (0.61, 0.92) |
| Weibull | 0.74 | (0.68, 0.79) |
| log-normal | 0.75 | (0.67, 0.85) |
| gamma | 0.75 | (0.68, 0.83) |
| generalized gamma | 0.72 | (0.68, 0.76) |
