# Supplementary material for: Community-level impacts of spatial repellents for control of diseases vectored by Aedes aegypti mosquitoes
Source: PLoS Comput Biol. 2020 Sep 25;16(9):e1008190. doi: 10.1371/journal.pcbi.1008190 (PMC7541056; doi:10.1371/journal.pcbi.1008190)
Supplement: S4 Table — (DOCX) [file pcbi.1008190.s012.docx]

**S4 Table. Survival functions.**

| Model | Probability density function (f(t)) | Survival function | Parameters | With covariates | Mean (95% Confidence interval: lower bound, higher bound) | AIC | | ΔAIC |
| --- | --- | --- | --- | --- | --- | --- | --- | --- |
| Exponential | $\lambda e^{-\lambda t}$ | $e^{-\lambda t}$ | *λ* =rate | $\lambda=\lambda\left( dose \right)$  $\lambda\left( dose \right)=\frac{1}{e^{\beta_{f,control}+\beta_{f,dose}}}$ | *β_control_=*2.94 (2.86, 3.02)  *β_1ow_=* -0.29(-0.49, -0.08)  *β_high_=* -0.63(-0.85, -0.41) | | 8626 | 1222 |
| Weibull | $v\lambda t^{v-1}e^{-\lambda t^{v}}$ | $e^{-\lambda t^{v}}$ | *λ*=rate  *v =* shape | $\lambda=\lambda\left( dose \right)$  $\lambda\left( dose \right)=e^{\beta_{f,control}+\beta_{f,dose}}$ | *ν =* 2.70 (2.57, 2.84)  *β_control_=*3.04 (3.01, 3.07)  *β_low_=*--0.31(-0.38, -0.23)  *β_high_=*-0.49(-0.57, -0.41) | | 7558 | 154 |
| Log-normal | $\frac{1}{x\sigma\sqrt{2\pi}}e^{-\frac{(\ln(t-\mu)^{2}}{2\sigma^{2}}}$ | $\frac{1}{2}+\frac{1}{2}erf\left[\frac{\ln\left(x-\mu\right)}{\sqrt{2\sigma}}\right]$ | *μ* = location  σ = scale | $\mu=\mu\left( dose \right)$  $\mu\left( dose \right)=e^{\beta_{f,control}+\beta_{f,dose}}$ | σ *=*0.58(0.55, 0.60)  *β_control_=*2.84 (2.80, 2.89)  *β_low_=*-0.28(-0.40, -0.16)  *β_high_=*-0.87(-0.99, -0.74) | | 8051 | 646 |
| Gamma | $\frac{\lambda\left(\lambda t\right)^{\kappa-1}e^{-\lambda t}}{\Gamma\left(\kappa\right)}$ | No closed form | *λ*=rate  κ *=* shape | $\lambda=\lambda\left( dose \right)$  $\lambda\left( dose \right)=\nu/{e^{\beta_{f,control}+\beta_{f,dose}}}$ | κ = 4.14 (3.83, 4.48)  *β_control_=* 0.22(0.20, 0.24)  *β_1ow_=*0.29(0.19, 0.39)  *β_high_=*0.63(0.52, 0.74) | | 7776 | 372 |
| Generalized Gamma | $\frac{\lambda v(\lambda t)^{v\kappa-1}e^{-(\lambda t)^{\nu}}}{\Gamma\left(\kappa\right)}$ | $\begin{matrix} 1-(\gamma\{\kappa,(\lambda t)^{v}\}/\Gamma(\kappa)) \\ \gamma(s,x)=\int_{0}^{x} t^{s-1}e^{-t}dt \end{matrix}$ | *λ* =rate  *v* = shape 1  *κ* = shape 2 | $\lambda=\lambda\left( dose \right)$  $\lambda\left( dose \right)=e^{\beta_{f,control}+\beta_{f,dose}}$ | κ = 0.21 (0.16, 0.26)  *ν =* 8.82 (7.38, 10.53)  *β_control_=* 29.46(28.64, 30.30)  *β_low_=*-0.33(-0.38, -0.28)  *β_control_=*-0.36(-0.42, -0.30) | | 7404 | 0 |
