# Supplementary material for: Community-level impacts of spatial repellents for control of diseases vectored by Aedes aegypti mosquitoes
Source: PLoS Comput Biol. 2020 Sep 25;16(9):e1008190. doi: 10.1371/journal.pcbi.1008190 (PMC7541056; doi:10.1371/journal.pcbi.1008190)
Supplement: Attachment — Submitted filename: SREffects_ReviewComments_PLOSCOMPBio.pdf [file pcbi.1008190.s013.pdf]

We like to thank the reviewers for thoughtful comments. Please find underneath a point-by-point response.

Reviewer's Responses to Questions

Reviewer #1:

The authors present a compelling framework to assess a broad range of public-health impact and product-development profiles through the interpretation of targeted small-scale entomological experiments combined with a rational model framework.

It is especially valuable that this paper takes a purposefully broad view of potential combinations of distinct spatial-repellent effects and maps out the relevance of different components and combinations of components on population-level effects -- including a thoughtful sensitivity analysis.

The only major concern is the handling of the fits in Figure 2, particularly the mismatch between the chosen functional forms and what is realized in the control experiments. This is mentioned in the text (L161-166) and explored to some degree in S1 Table, but unlike for survival -- which has a graphical exploration (S1 Fig) and quantitative assessment of fit quality (S3 Table) -- the sensitivity of downstream results to interpretation of blood-fed data remains a bit unclear.

1a. At a minimum, another supplemental figure demonstrating the fit quality of the scenarios in S1 Table in relation to Figure 2 would be appreciated. **We added the corresponding figure (S1 Fig)**

1b. It might also be a useful activity to explore scenarios where some fraction of experimental mosquitoes (~30%) will never feed, where not all partial feeds are "non-absorbing" state (~10%), or where mosquito feeding is a bi-modal phenomenon (first ~40% feed within hours, next ~20% feed over days). **As per the reviewers suggestion, we added a couple of scenarios to explore the SR effects on blood feeding in more detail. We show that, while these more complex models improve the model fit (based on AIC) by better capturing the early biting behavior, the general dose effect is relatively robust across model estimates. We added S2 Figure, S2 Table, and the following text to illustrate this:**

"Further, when expanding the model to include instantaneous feeding and a probability of never feeding, we find, based on AIC, that these models indeed better fit the data (S2 Table, S2 Figure). Specifically, mosquitoes exposed to the control product were more likely to feed directly (62%) than when exposed to a low (42%) or high (20%) dosage regimen. Mosquitoes under control or low scenarios are estimated to feed eventually, whereas 39% of high exposed mosquitoes are expected to never feed whatsoever. When comparing the average time at which 50% of mosquitoes have fed, the general impact of the SR is relatively robust to the different model assumptions. Treatment effects are estimated to be somewhat larger under the more complex models (S2 Table). (lines 173-182)"

Minor comments:

2. There introduction of model parameters and the mathematical symbols feels a bit informal in

some places. For example, referring to "o=Relative gonotrophic cycle length" in Figure 1, "delayed oviposition" on L134, and "oviposition rate" on L160.

We checked the manuscript for consistency and made the proposed adjustment throughout.

3. A more accurate formatting of highest density intervals might also include a % symbol after the ranges (where appropriate).

We made the proposed changes throughout the manuscript

4. The horizontal axis of Figure 2 would be easier to interpret if the tick marks and labels were in hours, e.g. 6h, 12h, 18h.

We made the proposed changes to Figure 2.

5. Figure 4: If it is intended that related off-diagonal columns can be compared by eye between the two dosages, this is a challenge with the current design choice to use the above and below diagonal halves in this way.

We made the proposed changes to Figure 4.

6. L282 and S4 Fig: Where do the univariate sensitivity ranges come from and how much do those choices impact the apparently most sensitive variables? **We used ranges from literature for those parameters that have empirical grounding or have been explored in other modeling studies. For parameters that reflect a probability or proportion, we explored values from 0 to 1. For parameters that are context specific, we explored an order of magnitude higher and lower. We adjusted this for S6 Fig J, which reflected two orders of magnitude earlier. The interpretation remains unchanged.**

7. L338: "chemical actives" --> "active chemicals"? **We made the suggested change throughout in the text.**

8. L369: "per see" --> "per se" (italic) **We made the suggested change in the text.**

9. L541: Is this a robust assumption, or does the relationship of adult densities to oviposition rates have some uncertainty in larval density-dependent dynamics? **This assumption is robust assuming breeding sites to be sufficiently abundant to not approach carrying capacity. We added text to highlight this assumption:**

" Assuming abundant breeding sites and thus limited impact of density dependent processes, a general form for equilibrium mosquito density is $m = \varepsilon / g$, where ε is the rate of emergence of new adult mosquitoes and g is death rate. **(lines 561-564)"**

10a. The companion paper (ref #25 in Parasites & Vectors) has a reference now, it seems, so this submission should be updated accordingly. **We made the suggested changed to the reference list.**

10b.If journal restrictions do not prevent it, the current manuscript would certainly benefit from some reproduction of Figure 6 in the supplement. **We added Figure 6 to the supplement (see S7 Fig)**

Reviewer #2

The manuscript presents a mathematical model of the impact of spatial repellents on the force of infection of dengue. The model is based on laboratory and semi-field research with *Aedes aegypti.* Model parameters included direct mortality, but also sublethal effects of spatial repellents on host-seeking, blood feeding, and flight. I have the following comments.

General comments. There is no question that the manuscript represents a very large research effort. The manuscript, however, is very tedious to read and the writing is often unclear (see specific comments below). Additionally, I have some concerns about the methodology.

1. The Abstract (lines 21-22) and Figure 1 indicate that the model is based on the premise that mosquito production occurs outdoors forcing host-seeking females to enter houses to acquire a blood meal. However, in the semi-field experiments conducted in Iquitos, Peru, mosquitoes were released within houses. Authors please respond to this concern.

Reproduction is modeled to be independent of the location of the mosquitoes but rather depends on the length of the gonotrophic cycle of mosquitoes. This may be affected by SRs. We have altered text to clarify this:

"While both o_C and g_C are affected by the time spent indoors and outdoors, we make no assumptions on where oviposition takes place. Oviposition therefore has no effect on the where mosquitoes spend their time spent and vice versa. " (lines 568-571).

Results from the analyses on semi-field experiments are independent of reproduction rates. The reviewer is correct that, contrary to the model framework, mosquitoes in the semi-field experiment are repelled from entering a new house from another house rather than from outside. However: " For this framework, the main interest lies in relative effects of product exposure."(lines 388-389). We expect the relative differences between dose effects to be similar for mosquitoes entering houses from neighboring premises or outside. We have clarified this assumption in this in the text:

"Here, we assume that the relative effect of ρ is similar for mosquitoes being repelled from outside as from entering from a neighboring space, even though the overall entry rate might be different between these two scenarios." (Lines 735-737)

2. Laboratory experiments were conducted with a pyrethroid "resistant" strain of Aedes aegypti, but field experiments were carried out using a "susceptible" strain. No data on the toxicity of transfluthrin or other pyrethroid insecticides to either strain is presented to support these characterizations. I am concerned that the response of resistant and susceptible mosquitoes to transfluthrin would be different so that some parameters would be over/under estimated resulting in an erroneous model. Authors please comment on this concern. **We have added background information on the toxicity essays (lines 612-613). Further, the reviewer is right that, apart from using different means of application or delivery of the product, the use of different mosquito populations is another limiting factor of this approach. We have added text to the introduction to highlight that this exercise is not meant as a formal projection of a single products impact:**

"Because these experiments used differing means of application or delivery of transfluthrin and mosquito populations with different levels of resistance to pyrethroids, this work does not constitute a projection of the epidemiological impact of a singular product formulation. However,

these data demonstrate how this model can be used to estimate the relative change in DENV force of infection brought about by a product with such properties introduced into a community at a given coverage." (Lines 125-131)

3. Additionally, I am concerned about the use of an artificial membrane system to evaluate host-seeking and blood-feeding by SR-exposed mosquitoes. It has been my experience that mosquitoes are less responsive to an in vitro system than to a human forearm. If the lab strain was verified to be pathogen-free, why weren't the mosquitoes allowed to feed on a human host?

While it may be true that a human arm is more attractive to a host-seeking mosquito than an artificial membrane feeding system (which arguably is dependent on the human host itself too), most important for the current study is that a matched control was used during *in vitro* experiments to compare *Aedes aegypti* feeding responses between SR unexposed and exposed test populations. The parameter fitted from these data reflects the relative difference between the exposed and unexposed populations and do not reflect the absolute biting rates. We agree with the reviewer that, if the latter were to be the goal, experiments with mosquitoes feeding on human hosts would be preferred.

Specific comments.
1. Abstract, lines 26-27. "…chemical's lethal effect but delayed biting and associated negative feedbacks on the mosquito population…" Please clarify what is meant by "associated negative feedbacks".

Here, we are referring to impacts on the mosquito population sizes if mosquitoes, over the duration of their lifetime, have fewer oviposition events. We clarified this in the abstract:

"Our results indicate that the greatest impact on force of infection is expected to derive from the chemical's lethal effect but delayed biting and the negative effect this may have on the mosquito population could elicit substantial impact in the absence of lethality. (lines 25-28)"

2. Lines 29-31. Please be specific about what is meant by "potential adverse impacts". **We clarified this sentence:**

"We also demonstrate that, through an increase in the number of potentially mosquito bites, increased partial blood-feeding and reduced exiting may illicit adverse impacts, which could offset gains achieved by other effects. (lines 29-31)"

3. Authors' summary. Lines 43-46. "…negative feedbacks on the mosquito population, elicit its own substantial impact." Again, the meaning of this statement is not clear. **We clarified this sentence:**

"We show that, while product-induced lethality accounts for the majority of the product's impact, delayed blood feeding can, through its negative impact on mosquito population sizes, elicit its own substantial impact. (lines 43-46)"

4. Lines 46-47. "Adverse effects of increased partial blood-feeding and reduced exiting could offset gains achieved by other effects." Please be specific about the "other effects". **We clarified this sentence:**

"Adverse effects of increased partial blood-feeding and reduced exiting could offset gains achieved by other effects such delayed blood feeding and lethality. (lines 46-47)"

5. Introduction, lines 54-57. "…more effective..." You just stated that there were setbacks in development of effective vaccines, suggesting that vaccines were not effective.

The setbacks pertain to limited efficacy for some but not all serotypes and the potential for adverse effects.

6. Lines 72-75. "…can promote adverse behaviors…" Why would these behaviors be adverse if the end result is successful blood feeding and oviposition? **We clarified this sentence:**

"In addition, excitatory effects can promote unintended behaviors, such as reduced exiting or increased probing, wherein hyper-agitated mosquitoes remain in treated spaces and attempt multiple, partial blood meals prior to full engorgement and oviposition (1). (lines 73-76)"

7. Lines 116-119. "…distinguishing probing from time until oviposition." These are two different behaviors and it is not clear how they are connected. Do the authors mean "blood feeding" rather than "probing"? **This phrasing was indeed confusing. We clarified this sentence:**

"Our model considers a broad range of acute and delayed effects, including repellency (decreased entry) and expellency (increased exit), excitatory effects on biting, decoupling biting rates from time until oviposition by accounting for both full and partial blood meals, and examining the context specificity of epidemiological impact. (lines 117-121)"

8. Line 120. "…the product effect is hereby referred to as 'lethality'…" So the myriad effects that the authors attribute to spatial repellents are included in one parameter "lethality"?

We here refer to the direct toxic effect of the product, which we refer to as 'lethality' and is measured in the longevity experiments. This is captured by two parameters: acute lethality and delayed lethality (explained in lines 179-183).

9. Line 121. "…hazards due to transit…" Authors please consider rephrasing this. The manuscript involves entomology not engineering. **We rephrased this wording:**

"..hazards experienced when moving from one house to another.. (lines 123-124)"

10. Results. Lines 150-153. "This result was largely driven by a reduction in the rate at which full blood meals were taken, with the average time until a full blood meal increasing relative to the control by 46% (HDI: 27-65) (low) and 74% (HDI: 50-100) (high)." Does the reduction also result from delays in successful host-seeking? **Yes, we believe that reduced biting rates in part may be attributable to interference with host-seeking (also discussed in introduction, lines 61-64). Additional experiments would be required to explore whether this, or other mechanisms drive the prolonged time until blood feeding.**

11. Line 154. "…partial blood meals (probing effect)…" Are the authors suggesting that partial blood meal result from aberrant probing? **We clarified the phrasing:**

"... partial blood meals due to incomplete imbibing and/or aberrant probing... (lines 159-160)"

12. Lines 156-157. "…increased insignificantly relative to the control in response to low exposure by 5% (HDI: -19-26) but did increase after high exposure 28%..." Was the 28% increase statistically significant? **This effect was indeed significant. We added this to the text.**

13. Line 207. "Repellency was lower at the higher dosage of transfluthrin…" A counter intuitive finding that the authors should explain in the discussion section. **We have added this to the discussion section:**

"Irritancy, inducing effects such as increased partial blood feeding and repellency, was common at low, sub-lethal dosages (13) and could affect the potential net benefit of a SR product. This finding is consistent with earlier work showing that the modes of action of active chemicals depend on the dosage of the product, with irritancy being more prevalent at lower dosages (13,22). (lines 343-347)"

14. Line 283-284. "We found that an SR product with characteristics similar to those in our model were less effective if the EIP (n) were longer and baseline mortality rates (gU) were higher." Don't the sublethal effects of SRs increase the EIP? **No assumptions are made on SR affecting the duration of the EIP.**

It is not clear why a high baseline mortality rate leads to a decline in the effectiveness of SR products.

Under these assumptions, only a small proportion of mosquitoes survives long enough to become infectious, resulting in a low baseline FOI. Further increasing the mortality would have a relatively small impact on FOI.

15. Discussion, line 325. "…increased feeding-induced mortality…" Please explain what this means. Is there a supportive reference that the authors can cite? **We clarified this in the text and added references that examined this effect:**

"Conversely, an increase in number of feeds could be accompanied with increased mortality due to the risks involved with blood feeding (26-28)(lines 340-341)"

16. Lines 330-332. "How these effects attenuate as chemical actives decay over space and time…" Is this a potential weakness of an SR for prevention of mosquito bites and reducing the FOI? Unless an SR is continuously released then effects of the SR would be short lived because newly emerged adults would replace those impacted by the SR. Authors please comment on this.

As with any vector control intervention, there will be a period of efficacy (product life) afterwards which efficacy will wane until failure. This is a characteristic of product development/design. The key is to replace interventions when the application period falls below levels of efficacy. So, if a specific SR product life is one month then that product will need to be replaced at one month. We clarified this in the text:

"How these effects attenuate as active chemicals decay over space and time will also be critical to quantify, to enable better prediction of downstream effects on neighboring premises and to assess the product life and consequential replacement strategies. (lines 349-352)"

17. Line 349. "…repellency effect could protect mosquitoes from entering a house…" Do the authors mean "…could prevent mosquitoes from entering a house…"? **We adjusted the text as suggested.**

18. Lines 353-355. 'The potential for diversion is strongly tied to the risks a mosquito experiences both from its exposure to the product and from its transit between houses." How can "transit between houses" be a cause of diversion? **This sentence is referring to the risks experienced during transit, i.e., if hazards of transiting are substantial, the chances of surviving the move from one house to the other are lower and the risk of diversion will be too. This is clarified in the text:**

"The potential for diversion is strongly tied to the risks a mosquito experiences from its exposure to the product and from the hazards a mosquito experiences when moving from one house to the other. In settings where transit is relatively hazardous, SRs that reduce time indoors are expected to have a greater impact. (lines 372-376)"

19. Lines 366-370. "Estimated relative effects were robust to different assumptions on time-varying hazards, justifying our simplifying assumptions. In other words, while more detailed models could provide a better fit to the experimental data, these would in part capture experimental artifacts that are not per see [sic] reflective of a natural response to product exposure." These sentences are poorly constructed and not informative. What artifacts are the authors referring to?

The meant artifacts are described here:

"Capturing realistic effects of product exposure in laboratory experiments is not straightforward. Exposure in the used experiments was short, yet continuous, resulting in a strong effect shortly after exposure. In the field, longer term, intermittent exposures are more likely and are expected to illicit a more gradual effect on mosquito-bionomics. The latter would more closely resemble model assumptions that rates and product effects are on average constant over time. For this framework, the main interest lies in relative effects of product exposure. (lines 383-389)"

20. Materials and Methods. Lines 435-439. The authors are hedging on the validity of their model. They should speculate on how the difference in application methods would have affected their model.

The reviewer is referring to the following lines:
"The experiments comprising the second component of our study all involved transfluthrin but did so with differing means of application or delivery of that chemical. Consequently, the third component of our study should be regarded as a hypothetical, but empirically grounded, analysis rather than a specific analysis of a singular product formulation."

This statement is to highlight that we do not aim to present a model parameterized for one specific optimized product but rather use the transfluthrin studies as an illustration. We added text to clarify why this is:

"As application methods may result in different levels of volatile particles that mosquitoes are exposed to, we cannot directly compare the relative product impacts on mosquito bionomics. (lines 460-462)"

21. Line 446. What is a "well-mixed community"? **We clarified this is in the text:**
"We assumed a well-mixed community meaning that each house has an equal probability of being visited by a mosquitoes. (Lines 470-472)"

22. Lines 447-450. This sentence is poorly constructed. "…assuming the possibility of repellency or attraction of the product…" Mosquitoes are attracted to an SR? **We agree this was confusing and removed the word 'attraction' from this sentence.**

23. Lines 491-493. "…a mosquito that is successfully repelled by the product may experience additional mortality later—e.g., due to predation—…" Use of "additional" suggests that "the mosquito" died previously. Can the authors cite a published study on predation as a significant mortality factor for Aedes aegypti? **We removed the word 'additional' as per the reviewer's suggestion. We added references estimating mortality risks indoors and outdoors and how this may affect disease dynamics. We agree with the reviewer that this are hard to estimate. The relative mortality indoors vs outdoors is therefore a free parameter that is explored in the sensitivity analysis.**

24. Lines 582-587. My concern about the use of resistant and susceptible mosquito strains is expressed above. **As described above, this is now addressed in more detail in the discussion.**

25. Lines 592-595. My concern about the use of an artificial feeding system is expressed above. **See above for discussion of this issue.**

26. Lines 616-618. "…contained transfluthrin-treated material or solvent alone…" Please describe the material used to release transfluthrin and the solvent. **We clarified this in the text:**
"The center hut contained cotton treated with transfluthrin or solvent alone (control, technical grade aceton) during baseline experiments. (lines 647-649)"

27. Line 626. "…three different treatments (control, low: 0.0025g/m2, high: 0.005g/m2)…" How were these release rates determined? **Chemical dosing in experiments was based on dosing for registered transfluthrin emanators, per guidance by manufacturer.**

28. Line 627. "(DOI 10.17605/OSF.IO/5HCPF)" What does this mean? **This is the link to the data repository. This has been clarified in the text.**

29. Figure 1. My comments about Figure 1 have been give above. **Addressed above**
30. Figure 2A, C. Control data appear to be poorly fit to the regression lines. Some measure of fit of the data points to the regression lines (e.g., R-square) should be given. In Figure 2C there is no regression line for the low dosage of transfluthrin. **We addressed the fits in more details in our response to reviewer #1. We included more detailed models that better capture the data. We compared the model fit of different model variants using AIC. Lastly, we illustrate the differences in interpretation of SR effects under different model variants and show that effect estimates are relatively robust to these different model assumptions. The low dosage regimen in Figure 2C overlaps with the high dosage regimen. We have added this to the legend.**

Reviewer #3: The authors present an interesting experimental approach to epidemic spreading and its sensitiveness to spatial repellents. The work is up to my knowledge original and results seem correct.

In a revised version I suggest the authors to consider the following two issues:
1) How can the modelling issues discussed in this paper be applied with real data? **With real data, we assume the reviewer means field data from for instance controlled trials of the product. We are indeed exploring if we can incorporate the presented model into an inference framework for estimating effects observed in the field. A specific example would be the results of the following randomized controlled trial:**
https://clinicaltrials.gov/ct2/show/NCT03553277?term=spatial+repellent&cntry=PE&draw=2&rank=1
This is clearly a complex exercise that would require careful design and close collaborations between modelers and field epidemiologists and entomologists. We added text to highlight this potential application:

"The presented framework could be used to analyze population-level studies on this and other vector control techniques that have multifaceted effects on the ecology and bionomics of mosquitoes. (Lines 378-380)"

2) Some recent works use agent-based models to investigate the impact of anti-malaria factors in epidemy spreading.
In Journal of Theoretical Biology 484 110030 e.g. an agent model is introduced for assessing the influence of drugs and gametocitemia periods in the spreading scenario, with a calibration using real data. Some discussion in this scope would strengthen the paper in my opinion.

We thank the reviewer for pointing out this interesting paper. Indeed a powerful work. The kind of data described in this paper would be difficult to obtain for dengue viruses, given the distinct epidemiological features of both diseases. As the first data from randomized controlled trials become available, efforts will be made to validate models based on trial data. This will also help calibrate models to a specific setting. We added text to the discussion:

"This would further allow for a more detailed site specific calibration. "(line 381)

Have all data underlying the figures and results presented in the manuscript been provided?
Large-scale datasets should be made available via a public repository as described in the *PLOS Computational Biology* data availability policy, and numerical data that underlies graphs or summary statistics should be provided in spreadsheet form as supporting information.

Reviewer #1: None

Reviewer #2: Yes

Reviewer #3: None

PLOS authors have the option to publish the peer review history of their article (what does this mean?). If published, this will include your full peer review and any attached files.

If you choose "no", your identity will remain anonymous but your review may still be made public.

Do you want your identity to be public for this peer review? For information about this choice, including consent withdrawal, please see our Privacy Policy.

Reviewer #1: Yes: Edward Wenger

Reviewer #2: Yes: Charles S. Apperson

Reviewer #3: No
