# Supplementary material for: Community-level impacts of spatial repellents for control of diseases vectored by Aedes aegypti mosquitoes
Source: PLoS Comput Biol. 2020 Sep 25;16(9):e1008190. doi: 10.1371/journal.pcbi.1008190 (PMC7541056; doi:10.1371/journal.pcbi.1008190)
Supplement: Attachment — Submitted filename: SREffects_ReviewResponse_PLOSCOMBIO_QAtenBosch.pdf [file pcbi.1008190.s014.pdf]

We like to thank the reviewers for thoughtful comments. Please find underneath a point-by-point response.

1. **Validation:**

Editor: I agree with both reviewers that this manuscript is improved. However, further discussion on the validation of the model should be added, including what sorts of (future collection of) data could be used to validate the model further.

Reviewer #2: The authors have suitably revised their manuscript. The manuscript describes a robust investigation. Hopefully, the authors' intent is to conduct an area-wide field study to validate their model.

Reviewer #3: This version is better than the previous one. Still, I do not think previous remarks on validation with empirical data .... I suggest the author to improve their manuscript in this scope.

Authors: We have added a paragraph on how the presented model framework could be validated in the field (lines 380-393):

"Field studies are needed to validate the presented framework and disentangle the bionomics most affected by interventions in real-world settings. From an entomological point of view, these evaluations should integrate the deployment of entry and exit traps [30-32] and measurements of mosquito abundance in treated and untreated places [8]. Mark-release-recapture studies in (semi-)field settings could further contribute to estimating the potential for mosquito diversion and the circumstances under which this may or may not be a concern. Further, studies aiming at estimating how bionomic effects impact FoI require combined entomological and epidemiological efforts. Large-scale, cluster-randomized trials that examine the epidemiological impacts of community-level roll out of SRs, on seroconversion and/or disease incidence, should be appended with entomological indices as described above [7, 8]. The presented framework is intended to be used to analyze such population-level studies and help pinpoint the bionomic effects that have the biggest impact on FoI or those that present barriers for impactful implementation. "

2. **Model assumptions:**

Editor: In addition, a discussion of the limitations of the model and its underlying assumptions should be included (e.g. in the discussion section).

Reviewer #3: This version is better than the previous one. ... the limitations (e.g. neglection of heterogeneous effects) were properly addressed. I suggest the author to improve their manuscript in this scope.

Authors: We have added an additional paragraph on model limitations pertaining to assumed homogeneity in transmission. This is in addition to the paragraph on spatial considerations. We further describe how more detailed agent-based models could be used to check the validity of the model and the impact of simplifications. (lines 411-424)

" The Ross-McDonald framework used has a number of limitations that may affect the outcome of SR implementation. Bites are modeled to be homogeneously distributed, assuming individuals to be equally attractive to mosquitoes and mosquitoes distributing their bites equally over time and across houses. Departures from homogeneous mixing can affect predictions of transmission dynamics and the impact of control in both directions (such as explored in [33-36]). Typically, homogeneous mixing results in more efficient transmission, because bites are well distributed over all individuals. More detailed model frameworks are beneficial for disentangling the impact of transmission heterogeneity. These could also be used to cross-validate more parsimonious and transparent frameworks, such as the one presented here. While differences in transmission are expected under more heterogeneous transmission scenarios, earlier studies comparing agent-based models with Ross-McDonald-like frameworks showed the latter to be adequate in revealing the relative synergistic effects of vector control (purpose of present study) and can do so in full transparency [37]. "
